# A decision model for enhancing reaction yield: Pd(II) and Pt(II) metal insertion to free porphyrin

**DOI:** 10.55730/1300-0527.3743

**Published:** 2025-04-19

**Authors:** Özay ÖZAYDIN, Emel ÖNAL

**Affiliations:** Department of Industrial Engineering, Faculty of Engineering, Dogus University, İstanbul, Turkiye

**Keywords:** Chemical synthesis, decision support, experimental design, AHP, sensor technology, yield optimization

## Abstract

Chemical synthesis experiments are crucial for developing new materials, yet their inherent complexity and variability often lead to suboptimal results, causing significant resource and time wastage. This study proposes a novel decision support model to enhance the success rate of macromolecule synthesis experiments for sensor applications. The model leverages principles of experimental design and decision-making. We identified synthesis alternatives through a literature review and expert consultations, narrowing it down to 6 alternatives for metal salts, 3 for reactant mole ratios, 5 for organic solvents, 3 for reaction temperatures, and 4 for reaction times. Considering the time and cost constraints, this selection process reduced the potential 1080 experiments to a manageable number. The model incorporates conflicting objectives such as yield, purity, cost, and time to maximize reaction yield. Using the analytical hierarchy process (AHP), the model assists decision makers in balancing these tradeoffs, thereby increasing the likelihood of experimental success. The validity of the model was confirmed by conducting 25 laboratory experiments and comparing the results with existing literature.

## 1. Introduction

There are diverse challenges associated with chemical synthesis due to the complexity of creating new compounds. These include molecular intricacy, precision, environmental impact, economic viability, reaction conditions, and scalability [1,2]. In pharmaceuticals [3,4] and materials science [5], synthesizing complex compounds requires high sensitivity and selectivity to minimize side reactions and waste. Traditional methods often involve hazardous reagents, necessitating greener alternatives. Economic and practical concerns arise from expensive starting materials, rare catalysts, or extreme reaction conditions. Advances in synthesis rely on discovering novel reactions and milder, more scalable processes [6]. Collaboration across biology, engineering, and computer science is essential to address these challenges, particularly when transitioning from laboratory to industrial-scale production.

Addressing these challenges in chemical synthesis demands ongoing research, innovation, and collaboration to develop more efficient, sustainable, and economically viable synthetic methods. The hierarchical nature of chemical synthesis and the necessity of multilayered decisions align with the analytical hierarchy process (AHP), a widely used multicriteria decision-making (MCDM) method.

AHP has been widely applied across industries for decision-making. In the energy sector, it has aided in selecting modernization strategies for coal-fired power plants [7]. In textiles, it has supported maintenance policy selection [8,9]. AHP has been used in supplier selection, including warehouse location in pharmaceuticals and supplier evaluation in manufacturing [10,11]. Applications also include product selection in pharmaceuticals, dispute analysis in construction [12], green supply chain management in manufacturing, vehicle selection in the automotive industry [13], and multicriteria assessment in steel spare parts [14]. AHP prioritizes resilient factors in textile supply chains [15].

## 2. Materials and methods

**Figure 1:**
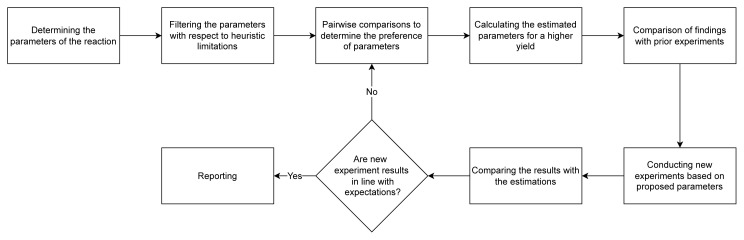
The framework of the study.

We took a systematic approach to optimizing chemical synthesis experiments using AHP. As illustrated in Figure 1, the process begins with determining the key parameters influencing the chemical reaction, including reactant mole ratio, organic solvent, reaction temperature, and reaction time.

Once these parameters were identified, they were filtered based on heuristic limitations including availability, cost, and practical feasibility, ensuring that only the most viable options were considered for further analysis. Next, we conducted pairwise comparisons using the AHP method to assess the relative importance of each parameter. This step involved comparing parameters in pairs to establish preferences and priorities. The preferences derived from these comparisons were then used to calculate the estimated parameters likely to yield the highest reaction efficiency, helping identify the optimal chemical synthesis conditions. Following this, the estimated parameters were compared with the results of prior experiments to validate their effectiveness and ensure alignment with existing empirical data. New experiments were then conducted using the proposed optimal parameters, testing the validity of the theoretical model in a real-world setting. The results from these new experiments were compared with the estimated parameters to check for consistency, verifying the accuracy of the AHP model. If the results met expectations, the process proceeds to the reporting section. If not, the parameters were reevaluated, and the process goes through the necessary steps. Finally, the successful findings and optimized parameters were documented and reported, and the methodology was completed by presenting the results and insights gained from the study. This structured approach ensured a thorough and systematic optimization of chemical synthesis experiments, leveraging both computational and experimental methods to achieve higher efficiency and success rates.

### 2.1. Decision model

**Figure 2:**
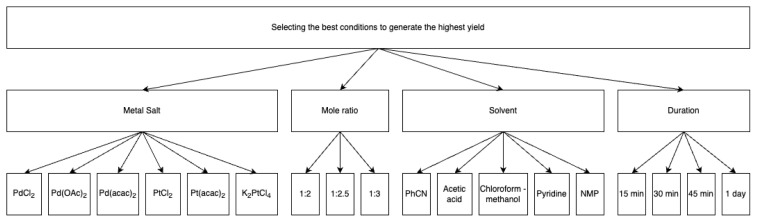
AHP model used in the study.

A comprehensive decision model was developed and implemented utilizing the AHP to optimize the yield of the chemical process under investigation. This methodological approach was selected for its capacity to systematically evaluate and quantify the relative importance of various factors influencing reaction outcomes. The AHP framework facilitates the decomposition of complex decision-making problems into a hierarchical structure, enabling a more nuanced analysis of overarching criteria and specific alternatives within each criterion. The primary objective of this model was to identify the optimal conditions for maximal yield attainment. By incorporating multiple levels of analysis, from broad categories to specific options, the model ensured a holistic approach to process optimization. This hierarchical structure allowed for the consideration of potential interactions between factors, thus providing a more comprehensive understanding of their collective impact on yield. The complete framework of this decision model is given in Figure 2, illustrating the hierarchical relationships and the range of alternatives considered for each criterion. This visual representation served as the foundation for the indepth analysis of the contribution of each factor to the overall yield optimization process. Through the AHP, quantitative weights were assigned to each criterion and alternative, facilitating a rigorous, data-driven approach to decision-making in this complex chemical system.

The decision hierarchy consisted of 4 primary criteria: metal salt, mole ratio, solvent, and reaction duration. Pairwise comparisons were conducted using Saaty’s 1–9 scale, where a score of 1 indicates equal importance, and 9 signifies that one criterion is more important than another. By going through each criterion in a pairwise manner, a pairwise comparison matrix was obtained. After normalizing the values and computing the priority vector, the final weight for each criterion was determined.

### 2.2. Metal salts

Metalloporphyrins are valuable for their enhanced molecular detection and optical properties. Nearly all metals have been incorporated into porphyrins over the past 50 years, as documented in numerous reviews [16–19]. Most are synthesized via metathesis, where a free-base porphyrin reacts with a metal salt, replacing pyrrole protons with a metal ion to form the metalloporphyrin and conjugated anion acid [20,21] as shown in Equation 1 and Scheme.


(1) $$\text{Por}-{\text{H}}_{2}+[{\text{M}}^{\text{II}}{\left(\text{anion}\right)}_{2}]\to [{\text{PorM}}^{\text{II}}]+2\;\text{anion}-\text{H}$$

The ionic radius of the central metal ion determines its compatibility with the porphyrin cavity that cannot easily accommodate ions larger than 2.01 Å [22]. Palladium (1.37 Å) and platinum (1.39 Å) fall within this limit, making them suitable candidates. However, while thermodynamically favorable, metal insertions are often kinetically hindered due to the rigidity of the porphyrin ligand and the properties of the metal ions, necessitating variable reaction conditions.

This study reviewed commercial metal salts, identifying 14 products—7 of which were Pd and the other 7 were Pt salts—that were prioritized based on the literature [16,17,23–25]. Commonly used salts PdCl_2_ and PtCl_2_ were initially selected. Considering budget constraints and yields reported in the literature, 6 economical salts (3 palladium and 3 platinum) were chosen [20,23,24,26,27].

### 2.3. Mole ratio

The effect of the metal ion on porphyrin mole ratio was examined, with studies showing that higher ratios (e.g., 6:1 or 10:1) enhanced reactant interaction but reduced complex formation efficiency, meaning excess metal ions and increased costs [28]. Conversely, minimizing the ratio to 1:3 optimized metalloporphyrin synthesis, balancing yield and resource use [20,24]. Based on this, mole ratios of 1:2, 1:2.5, and 1:3 were selected to maximize reaction efficiency while minimizing metal salt consumption.

### 2.4. Solvent

The choice of solvent is critical for ensuring mutual solubility between metal salts and free-based porphyrins. Reactions have been conducted in acidic, basic, and neutral media, each with challenges: excess metal salts in acidic conditions, solvent coordination in basic media, and competitive reactions in neutral solvents [16,17,23–25,29]. These methods often yield low efficiencies and require high temperatures (100–180 °C) and long reaction times. Based on the literature, we selected 5 high boiling point, noncoordinating, microwave (MW)-active solvents to optimize the process.

### 2.5. Duration

The slow metalation of inert metal ions into free-base porphyrins often results in low yields, purification challenges, and lengthy processes. MW heating offers a faster alternative, converting electromagnetic energy into heat through dielectric losses, enabling rapid and targeted heating [30]. However, it requires molecules with high polarity and dielectric constants, limiting its applicability [24]. N-methyl-2-pyrrolidinone (NMP), with its high polarity (6.7) and dielectric constant (32.2 at 25 °C), was used to dissolve free porphyrins and reduce reaction times from hours to minutes. MW experiments were conducted for 15, 30, and 45 min, while other solvents like benzonitrile (PhCN), acetic acid, pyridine, and chloroform-methanol required 24 h to ensure completion.

## 3. Results

**Figure 3:**
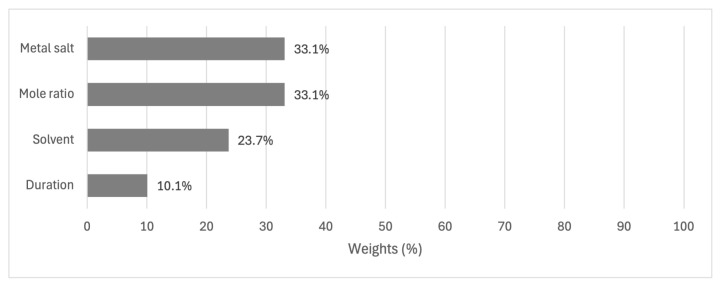
AHP weight results of the main criteria.

Our decision model for optimizing yield showed a nuanced interplay of factors, each contributing uniquely to the overall outcome. Equal importance was assigned to the choice of metal salt and mole ratio in the model, with each weighing 33.1% in the decision-making process. The equal weighting shows the critical nature of these 2 factors in determining the yield. The selection of solvent follows closely, accounting for 23.7% of the decision weight, while the duration of the reaction, though less impactful, still plays a significant role at 10.1%. These results are summarized in Figure 3.

**Figure 4:**
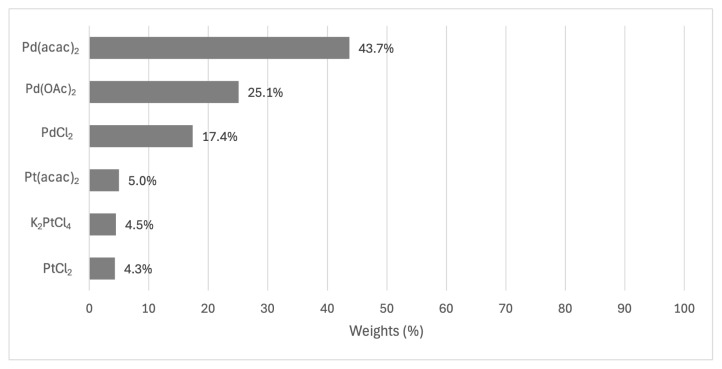
Results of alternatives for the metal salt criterion.

Regarding the specifics of each criterion, the choice of metal salt significantly influences the yield. Among the options considered, Pd(acac)_2_ was identified as the best choice, with a performance rating of 43.7%. This palladium-based salt substantially outperformed its nearest competitors, with Pd(OAc)_2_ following at 25.1% and PdCl_2_ at 17.3%. The marked superiority of palladium-based salts over their platinum counterparts was evident, with the best-performing platinum salt, Pt(acac)_2_, achieving only 5% performance. This stark contrast suggests that yield outcomes could be significantly enhanced by focusing on palladium-based salts, particularly Pd(acac)_2_. The results of metal salts are given in Figure 4.

**Figure 5:**
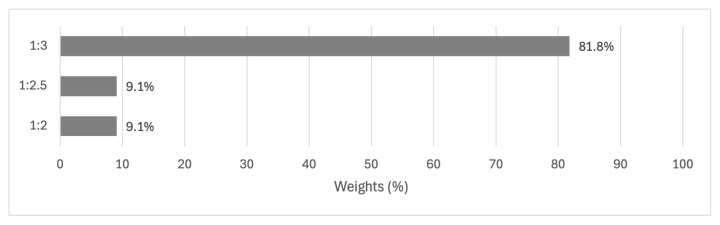
Results of alternatives for the mole ratio criterion.

The mole ratio was a critical factor with a clear optimal choice. The 1:3 ratio dramatically outperformed other options, with an 81.8% performance rating. This is contrasted sharply with the 1:2.5 and 1:2 ratios, both of which achieved only 9.1% performance. The substantial gap between the optimal ratio and the alternatives shows the importance of precisely controlling this aspect of the reaction conditions to maximize yield. The results are shown in Figure 5.

**Figure 6:**
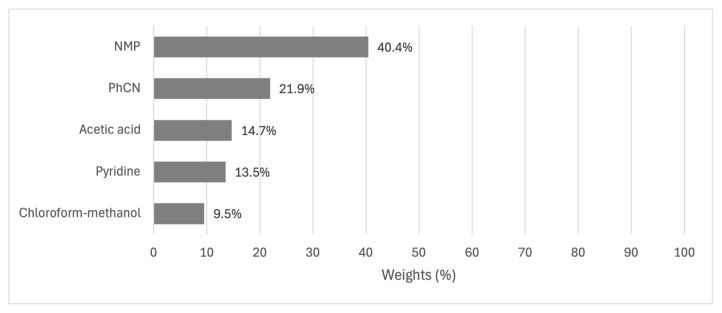
Results of alternatives for the solvent criterion.

Regarding solvents, NMP was identified as the most effective option, with a performance rating of 40.4%. It was better than the next best alternative PhCN that achieves 21.9% performance. The remaining options (acetic acid, pyridine, and chloroform-methanol) showed progressively lower performance. This distribution suggests that the choice of solvent can have a substantial impact on yield, with NMP offering a clear advantage over other options. The results of different solvents are given in Figure 6.

**Figure 7:**
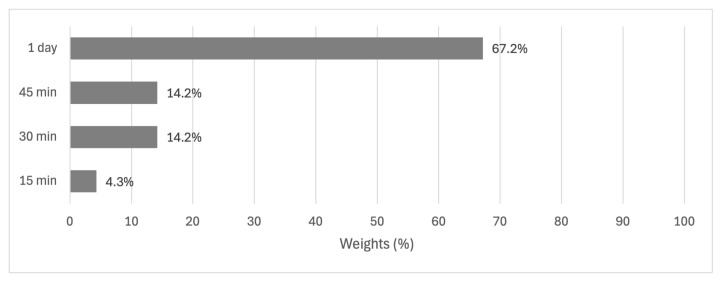
Results of alternatives for the duration criterion.

While the duration of the reaction was assigned the lowest weight in the decision model, its impact on yield should not be underestimated. The data shows a strong preference for longer reaction times, with a duration of 1 day achieving a 67.2% performance rating. This is starkly contrasted with shorter durations, with 45 and 30 min both reaching only 14.2% performance, and 15 min lagging further behind at 4.3%. These results, as shown in Figure 7, indicate that allowing sufficient time for the reaction to progress is crucial for maximizing yield despite the lower overall weight of this factor in the decision model.

Considering all criteria, the optimal conditions for achieving the highest yield involve the use of Pd(acac)_2_ as the metal salt, using a 1:3 mole ratio, using NMP as the solvent, and performing the reaction for 1 day. This combination leverages the top-performing option in each criterion, potentially leading to synergistic effects that could further enhance yield beyond what might be expected from considering each factor in isolation.

It is worth noting that while these conditions appear optimal based on the current data and model, the complex nature of chemical reactions means that interactions between these factors may not be fully captured in this analysis. Furthermore, practical considerations such as cost, availability of materials, and specific requirements of the reaction setup may influence the final choice of conditions in real-world applications. This decision model provides a robust framework for optimizing yield, offering clear guidance on the most influential factors and their optimal settings. By focusing on the use of Pd(acac)_2_, maintaining a 1:3 mole ratio, using NMP as the solvent, and allowing for extended reaction times, researchers and process engineers can significantly enhance their chances of achieving high yields in chemical processes.

## 4. Discussion

The first attempt to introduce Pd(II) into free-base porphyrin used a classical and well-known method [31] using PdCl_2_ in PhCN (bp: 191 °C), following a 2:1 mole ratio of metal to porphyrin for one day at reflux temperature. The reaction was completed with a 68% yield. When we applied MW heating, known to enhance the rate of metalation during synthesis [24], for 15 min and 45 min, arc formation occurred in the MW reaction system, resulting in a 0% yield. Thus, PdCl_2_ is not the best metal source when MW energy is used, since elemental Pd metal formation limits the completion of the reaction [20]. Other experiments that changed the solvent to acetic acid [23] and increased the mole ratio from 2 to 2.5 and 3 did not enhance the reaction yield (Table, rows 1–8). The maximum reaction yield of 15% was obtained by 3 equiv. of metal salt for 1 day was successful but insufficient. The 72% reaction yield in the literature [27] with Pd acetate was promising. Therefore, Pd acetate (Pd(OAc)_2_) salt was used instead of PdCl_2_ under the same conditions, but only a 5% yield was obtained. Despite working with the same conditions as in the literature, only a 24% yield was achieved (Table, rows 9–11). This can be explained by the fact that the porphyrin macrocycle on which the metallization reaction occurs has different substitution groups than in the literature.

However, a mole ratio of 2:1 metal salt to porphyrin resulted in high reaction yields (68%, Table, row 3). When the organic solvent was changed, a mole ratio of 3:1 was found to be optimal, so this mole ratio was used in all other trials. Thus, by determining the successful mole ratio parameter, fewer chemical experiments were required for solvent and time optimization. According to the literature, another method for the metalation of porphyrins is the use of metal acetylacetonate M(acac)_2_ complexes as the metal carrier. This carrier has desirable properties such as the ligand(s) in the metal carrier complex providing good solubility for the metal, especially in weak organic solvents. In addition, they permit ready displacement by the porphyrin, have a weak and volatile conjugate acid, and are readily available [32]. Further experiments were performed using pyridine as the solvent for the same reasons in Dean et al. [20]. Pyridine is more readily removed from the reaction product by (rotary) evaporation or aqueous acid extraction than PhCN. Also NMP has a high boiling point and is a MW-absorbing solvent with an excellent dielectric loss ɛ (8.85 at room temperature and 2450 MHz) [20,33,34]. While no yield was obtained in the trials using MW energy with pyridine as an organic solvent for 15 and 45 min, the reaction at reflux temperature for 1 day was successful with 49% yield (Table, row 16). Although an acceptably high yield was obtained, 1 day is a long reaction time. While dimethylformamide (DMF) and PhCN solvents used in classical methods under the same conditions did not yield results with the Pd(acac)_2_ salt, the most remarkable result was the reaction yield of 81% using NMP as an organic solvent for a short reaction time of only 15 min (Table, rows 12–17).

Kinetic parameter studies have shown that Pt complex formation is slower than Pd complexes [35]. Thus, Pt(II) insertion is typically achieved only through the use of the most rigorous methods, such as a long reaction time [16] that ends with low reaction yields [25]. For this reason, palladium metallization reactions were studied first, and after the most suitable conditions were determined, platinum metallization reactions were carried out by considering fewer but more effective conditions. Rows 18–25 in Table show that only 2 of the 8 palladium metallization reaction attempts were successful, and the reaction yields did not exceed 25%. Both of the successful palladium metallization reactions were with the Pt(acac)_2_ metal salt in a 3:1 ratio in NMP organic solvent using MW wave energy. Although our previous publication [36] did not include as much detail as in the current study, the same conclusion can be drawn. The best conditions were 3 equiv. Pd(acac)_2_/NMP/180 °C/MW 15 min for 81% yield of Por-Pd(II), and 3 equiv. Pt(acac)_2_/NMP/110 °C/MW 45 min for 25% yield of Por-Pt(II).

The AHP model facilitated a structured decision-making process, effectively evaluating multiple criteria such as metal salts, mole ratios, solvents, and reaction durations. This approach was crucial for optimizing the conditions for metalloporphyrin synthesis, particularly in identifying the optimal parameters to achieve the highest yield. The hierarchical structure of the model helped break down complex decisions into more manageable problems, enhancing clarity and focus on optimization. By providing a quantitative assessment of the relative importance of various factors, the AHP model enabled a data-driven approach to decision-making. For instance, the model assigned equal importance to the choice of metal salt and mole ratio, each weighing 33.1% in the decision-making process. The solvent and reaction duration followed with weights of 23.7% and 10.1%, respectively. This quantitative analysis was pivotal in determining the most influential factors on the reaction yield of metal insertion synthesis. The predictions of the AHP model were validated through laboratory experiments. The optimal conditions identified by the model, such as using Pd(acac)_2_ as the metal salt, an NMP as the solvent, and a reaction duration of 1 day, were tested and confirmed to yield high results. This empirical validation reinforced the reliability and accuracy of the model in optimizing chemical synthesis processes. According to experimental results, the best conditions were a 1:3 mole ratio of Pd(acac)_2_ to porphyrin, NMP as a solvent, and 15 min. reaction duration at 180 °C/MW with 81% reaction yield for Por-Pd(II). For Por-Pt(II), the best conditions were 1:3 mole ratio, NMP, and 45 min at 110 °C(MW) instead of conventional heating with 25% yield.

While the AHP model assigned only 10.1% weight to reaction duration, experimental findings showed that extending reaction time (e.g., to 1 day) significantly improved metalloporphyrin yield. This discrepancy arises because AHP prioritizes multiple criteria beyond just reaction yield, including economic feasibility and experimental efficiency.

During pairwise comparisons, expert judgment and literature analysis emphasized metal salt and mole ratio as the most influential factors, given their fundamental role in metal insertion reactions. Reaction time was assigned a lower weight because it was initially assumed that optimal conditions (e.g., solvent and reactant ratio) would yield efficient reactions even at shorter durations.

However, experimental validation suggests that prolonged reaction times compensate for kinetic barriers, particularly in Pt(II) insertions that are inherently slower than Pd(II). The AHP model could be further refined by incorporating empirical feedback loops that dynamically adjust the duration weight based on observed reaction kinetics. Future studies could explore a hybrid approach that combines AHP and experimental validation to fine tune model predictions more effectively.

## 5. Conclusion

This study successfully implemented the AHP model to optimize chemical synthesis, particularly in the metallization of porphyrins. Key findings include identifying optimal conditions for maximizing yield, with Pd(acac)_2_ as the metal salt, a 1:3 mole ratio, NMP as the solvent, and a reaction duration of 1 day. When using MW heating, these conditions led to an 81% yield for Por-Pd(II) and 25% for Por-Pt(II). The AHP model provided a structured, quantitative approach to decision-making, validating its predictions through empirical laboratory results, proving the robustness and practical applicability of the model in optimizing complex chemical processes. Optimized synthesis processes in industry can improve efficiency and costeffectiveness in sectors like pharmaceuticals, materials science, and sensor technology.

## Figures and Tables

**Scheme:**
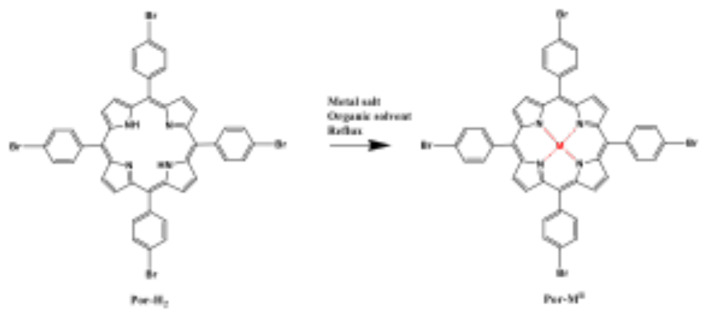
Synthesis of metalloporphyrin (Por-M^11^).

**Table:** Laboratory experiment conditions for Pd(II) and Pt(II) metal insertion to Por-H_2_.

#	MS	MR	Solvent	Temp.	Dur.	Condition	RY (%)
**1**	PdCl_2_	2	PhCN	191°C	15 m	MW	0
**2**	PdCl_2_	2	PhCN	191°C	45 m	MW	0
**3**	PdCl_2_	2	PhCN	191°C	24 h	Reflux	68
**4**	PdCl_2_	2	Acetic acid	RT	15 m	Stirring	0
**5**	PdCl_2_	2,5	Acetic acid	RT	15 m	Stirring	0
**6**	PdCl_2_	2,5	Acetic acid	RT	30 m	Stirring	10
**7**	PdCl_2_	3	Acetic acid	RT	30 m	Stirring	15
**8**	PdCl_2_	3	Acetic acid	RT	24 h	Stirring	20
**9**	Pd(OAc)_2_	2,5	Acetic acid	RT	30 m	Stirring	5
**10**	Pd(OAc)_2_	3	Acetic acid	RT	30 m	Stirring	5
**11**	Pd(OAc)_2_	3	Chloroform-Methanol	70°C	45 m	Stirring	24
**12**	Pd(acac)_2_	3	PhCN	191°C	15 m	MW	0
**13**	Pd(acac)_2_	3	DMF	153°C	15 m	MW	0
**14**	Pd(acac)_2_	3	Pyridine	180°C	15 m	Reflux	0
**15**	Pd(acac)_2_	3	Pyridine	180°C	45 m	Reflux	0
**16**	Pd(acac)_2_	3	Pyridine	180°C	24 h	Reflux	49
**17**	Pd(acac)_2_	3	NMP	180°C	15 m	MW	81
**18**	PtCl_2_	3	PhCN	191°C	24 h	Reflux	0
**19**	Pt(acac)_2_	3	PhCN	191°C	24 h	Reflux	23
**20**	Pt(acac)_2_	3	PhCN	191°C	15 m	MW	0
**21**	Pt(acac)_2_	3	PhCN	191°C	45 m	MW	0
**22**	Pt(acac)_2_	3	Chloroform-Methanol	70°C	45 m	Reflux	0
**23**	Pt(acac)_2_	3	Chloroform-Methanol	70°C	24 h	Reflux	0
**24**	Pt(acac)_2_	3	NMP	110°C	45 m	MW	25
**25**	K_2_PtCl_4_	3	NMP	110°C	45 m	MW	0
